# (*E*)-1-[4-(Prop-2-yn-1-yl­oxy)phen­yl]-3-(3,4,5-trimeth­oxy­phen­yl)prop-2-en-1-one

**DOI:** 10.1107/S1600536810031193

**Published:** 2010-08-11

**Authors:** S. Ranjith, A. Thirunarayanan, S. Raja, P. Rajakumar, A. SubbiahPandi

**Affiliations:** a Department of Physics, Presidency College (Autonomous), Chennai 600 005, India; b Department of Organic Chemistry, University of Madras, Guindy Campus, Chennai 600 025, India

## Abstract

The mol­ecule of the title chalcone derivative, C_21_H_20_O_5_, consists of two substituted aromatic rings bridged by a prop-2-en-1-one group. The dihedral angle between the two benzene rings is 28.7 (7)°. In the crystal, mol­ecules are linked into *C*(10) chains running along the *a* axis by inter­molecular C—H⋯O hydrogen bonds, and the chains are cross-linked *via* C—H⋯π inter­actions.

## Related literature

For the biological activity of chalcones, see: Di Carlo *et al.* (1999 ▶); Rao *et al.* (2004 ▶); Sabzevari *et al.* (2004 ▶); Litkei (1979 ▶); Pandey *et al.* (2005 ▶); Lawrence *et al.* (2001 ▶); Lin *et al.* (2002 ▶). For related structures, see: Suwunwong *et al.* (2009 ▶); Wu *et al.* (2005 ▶). For hydrogen-bond motifs, see: Bernstein *et al.* (1995 ▶).
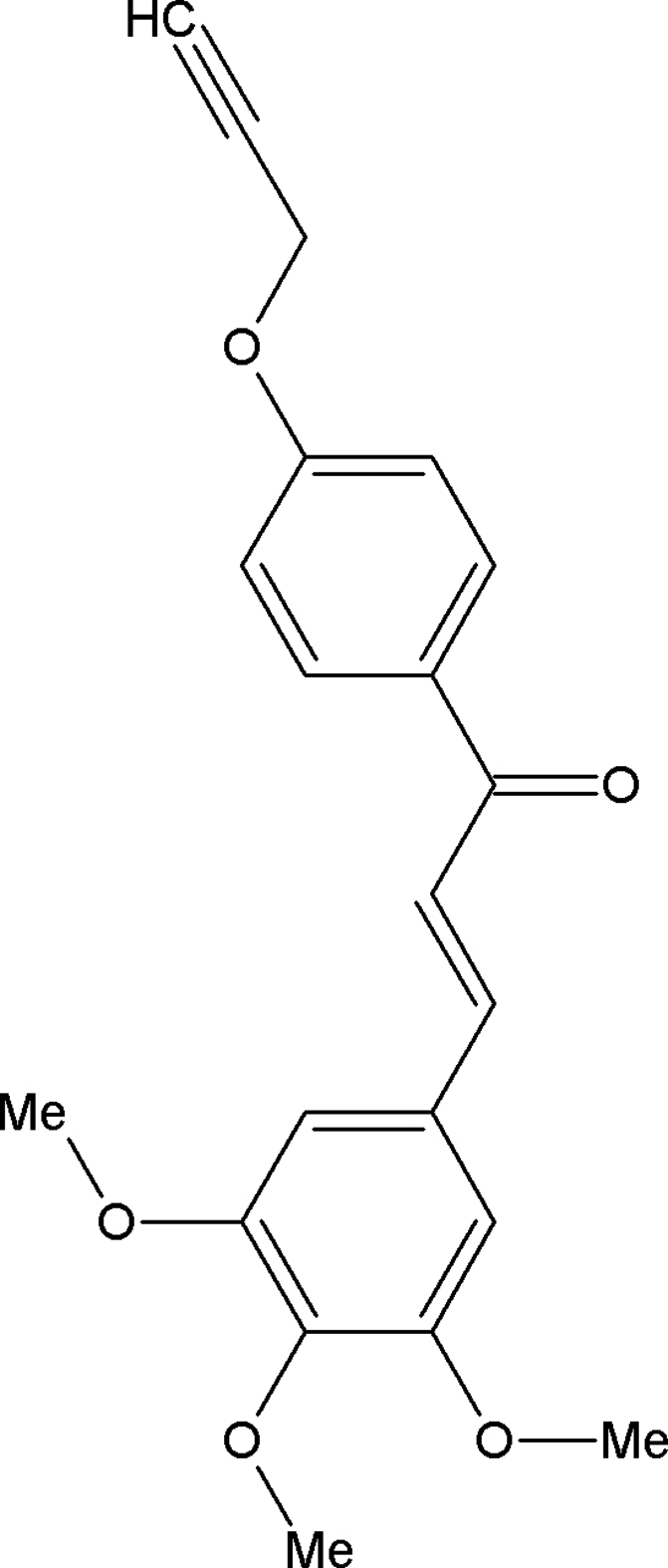


## Experimental

### 

#### Crystal data

C_21_H_20_O_5_
*M* *_r_* = 352.37
Monoclinic, 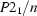

*a* = 11.6344 (8) Å
*b* = 11.5970 (7) Å
*c* = 14.4169 (12) Å
β = 107.763 (5)°
*V* = 1852.5 (2) Å^3^
*Z* = 4
Mo *K*α radiation
μ = 0.09 mm^−1^
*T* = 293 K
0.25 × 0.22 × 0.19 mm

#### Data collection

Bruker APEXII CCD area-detector diffractometer
Absorption correction: multi-scan (*SADABS*; Sheldrick, 1996 ▶) *T*_min_ = 0.981, *T*_max_ = 0.985
17592 measured reflections
4556 independent reflections
3382 reflections with *I* > 2σ(*I*)
*R*_int_ = 0.023

#### Refinement

*R*[*F* ^2^ > 2σ(*F* ^2^)] = 0.043
*wR*(*F* ^2^) = 0.128
*S* = 1.03
4556 reflections
242 parameters
H atoms treated by a mixture of independent and constrained refinement
Δρ_max_ = 0.23 e Å^−3^
Δρ_min_ = −0.19 e Å^−3^

### 

Data collection: *APEX2* (Bruker, 2004 ▶); cell refinement: *SAINT* (Bruker, 2004 ▶); data reduction: *SAINT*; program(s) used to solve structure: *SHELXS97* (Sheldrick, 2008 ▶); program(s) used to refine structure: *SHELXL97* (Sheldrick, 2008 ▶); molecular graphics: *ORTEP-3* (Farrugia, 1997 ▶); software used to prepare material for publication: *SHELXL97* and *PLATON* (Spek, 2009 ▶).

## Supplementary Material

Crystal structure: contains datablocks global, I. DOI: 10.1107/S1600536810031193/bt5297sup1.cif

Structure factors: contains datablocks I. DOI: 10.1107/S1600536810031193/bt5297Isup2.hkl

Additional supplementary materials:  crystallographic information; 3D view; checkCIF report

## Figures and Tables

**Table 1:** Hydrogen-bond geometry (Å, °) *Cg*1 is the centroid of the C13–C18 ring.

*D*—H⋯*A*	*D*—H	H⋯*A*	*D*⋯*A*	*D*—H⋯*A*
C19—H19*A*⋯O2^i^	0.96	2.48	3.396 (2)	161
C20—H20*B*⋯*Cg*1^ii^	0.96	2.61	3.487 (2)	152

Symmetry codes: (i) 
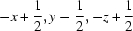
; (ii) 
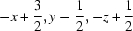
.

**Fig. 1.**
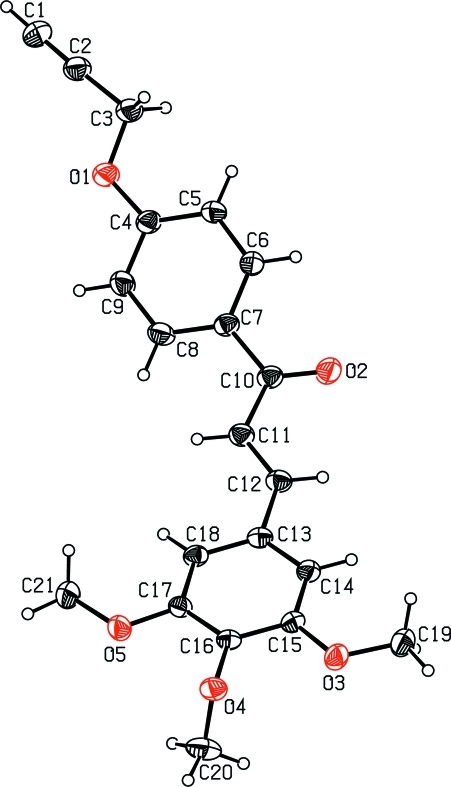
The structure of showing the atom-numbering scheme and intramolecular hydrogen bond. Displacement ellipsoids are drawn at the 30% probability level.

**Fig. 2.**
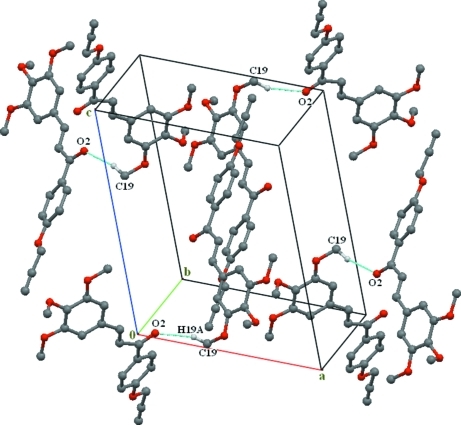
The molecular packing viewed down the b axis. Dashed lines shows the intermolecular C–H···O hydrogen bonds.

## supplementary crystallographic information

### Comment

Chalcones are one of the major classes of natural products with widespread distribution in fruits, vegetables, spices, tea and soy based foodstuff have recently been subjects of great interest for their interesting pharmacological activities (Di Carlo *et al.*, 1999). Chalcones are biosynthesized by plants, and an impressive number have been found toxic to cancer cells (Rao *et al.*, 2004; Sabzevari *et al.*, 2004). Chalcone epoxides have long been suspected as intermediates in the biosynthesis of plant flavonoids (Litkei, 1979). Chalcones can be easily obtained from the aldol condensation of aromatic aldehydes and aromatic ketones. This class of compounds presents interesting biological properties such as cytotoxicity (Pandey *et al.*, 2005), antiherpes activity, antitumour activity and may be useful for the chemotherapy of leishmaniasis among others (Lawrence *et al.*, 2001). Chalcones and flavonoids as anti-tuberculosis agents are also reported (Lin *et al.*, 2002). Against this background, and in order to obtain detailed information on molecular conformations in the solid state, an X-ray study of the title compound was carried out.

X-Ray analysis confirms the molecular structure and atom connectivity as illustrated in Fig. 1. The bond distances are of normal values and are comparable with the closely related structures (Suwunwong *et al.*, 2009; Wu *et al.*, 2005). The molecule of the title chalcone derivative (Fig. 1) exists in an E configuration with respect to the C11—C12 double bond [1.323 (2) Å] with torsion angle C10—C11—C12—C13 = 172.4 (1)°. The whole molecule is not planar as the dihedral angle between the two phenyl rings is 28.7 (7)°. The propenone unit (C10—C12/O2) is nearly planar with the torsion angle O2—C10—C11—C12= -0.9 (2)°. Atoms O2, C7, C10, C11 and C12 lie on the same plane with the most deviation of -0.023 (1)Å for atom C10. The mean plane through O2/C7/C10/C11/C12 makes interplanar angles of 19.7 (8)° and 14.5 (7)° with the planes of the two phenyl rings, respectively. The atoms O1, O3, O4 and O5 deviate by 0.044 (1), 0.014 (1), 0.043 (1) and 0.012 (1) Å, respectively, from the plane of the attached phenyl rings.

In the solid state, the title molecule is characterized by an intramolecular C12–H12···O2 hydrogen bond in which the carbon atom acts as a donor to the adjacent keto O atom. This hydrogen bond is responsible for the coplanarity of the C4—C9 benzene ring with the central propenone chain. This hydrogen bond completes a five-membered ring, which generates an S(5) motif (Bernstein *et al.*,1995). The atom C19 acts as a donor to the atom O2 of the neighbour molecule at (-*x* + 1/2, *y* - 1/2, -*z* + 1/2). This hydrogen bond is involved in a motif C(10) forming a chain along *a* axis. In addition, the crystal packing is stabilized by a C–H···π interaction between one of the methyl H atoms (H20B) and the centroid (cg1) of the C13–C18 ring (Table 1).

### Experimental

Compound was prepared through condensation of 4-hydroxyacetophenone (5 mmol, 1.57 g) with 3,4,5-trimethoxybenzaldehyde (5 mmol, 0.68 g) in 10% NaOH solution (1 ml), stirred at room temperature for 12 h (yield 65%, m.p. 146°C). The reaction mixture was poured into ice water (100 ml) and filtered. After the usual work-up, the product was purified by column chromatography. Further the corresponding phenol (2.0 g, 6.36 mmol) propargyl bromide (7.96 mmol) and anhydrous potassium carbonate (31.8 mmol) in dry DMF (15 ml) was stirred at 60°C for 24 h. The reaction mixture was then allowed to cool at room temperature and poured into ice water. The resulting precipitate was filtered, washed thoroughly with water and dissolved in CHCl_3_ (150 ml). The organic layer was seperated, washed with brine (1x150 ml), dried (anhydrous Na_2_SO_4_) and evaporated to give the crude dendron. Crystals suitable for X-ray diffraction were obtained by slow evaporation of a 95% chloroform solution.

### Refinement

All H atoms were fixed geometrically and allowed to ride on their parent C atoms, with C—H distances fixed in the range 0.93–0.97 Å and with *U*_iso_(H) = 1.5*U*_eq_(C) for methyl H 1.2*U*_eq_(C) for other H atoms.

### Crystal data

**Table:**

C_21_H_20_O_5_	*F*(000) = 744
*M**_r_* = 352.37	*D*_x_ = 1.263 Mg m^−^^3^
Monoclinic, *P*2_1_/*n*	Mo *K*α radiation, λ = 0.71073 Å
Hall symbol: -P 2yn	Cell parameters from 4556 reflections
*a* = 11.6344 (8) Å	θ = 2.0–28.3°
*b* = 11.5970 (7) Å	µ = 0.09 mm^−^^1^
*c* = 14.4169 (12) Å	*T* = 293 K
β = 107.763 (5)°	Block, white crystalline
*V* = 1852.5 (2) Å^3^	0.25 × 0.22 × 0.19 mm
*Z* = 4	

### Data collection

**Table:**

Bruker APEXII CCD area-detector diffractometer	4556 independent reflections
Radiation source: fine-focus sealed tube	3382 reflections with *I* > 2σ(*I*)
graphite	*R*_int_ = 0.023
ω and φ scans	θ_max_ = 28.3°, θ_min_ = 2.0°
Absorption correction: multi-scan (*SADABS*; Sheldrick, 1996)	*h* = −13→15
*T*_min_ = 0.981, *T*_max_ = 0.985	*k* = −13→15
17592 measured reflections	*l* = −19→14

### Refinement

**Table:**

Refinement on *F*^2^	Primary atom site location: structure-invariant direct methods
Least-squares matrix: full	Secondary atom site location: difference Fourier map
*R*[*F*^2^ > 2σ(*F*^2^)] = 0.043	Hydrogen site location: inferred from neighbouring sites
*wR*(*F*^2^) = 0.128	H atoms treated by a mixture of independent and constrained refinement
*S* = 1.03	*w* = 1/[σ^2^(*F*_o_^2^) + (0.061*P*)^2^ + 0.372*P*] where *P* = (*F*_o_^2^ + 2*F*_c_^2^)/3
4556 reflections	(Δ/σ)_max_ < 0.001
242 parameters	Δρ_max_ = 0.23 e Å^−^^3^
0 restraints	Δρ_min_ = −0.19 e Å^−^^3^

### Special details

**Table:**

Geometry. All e.s.d.'s (except the e.s.d. in the dihedral angle between two l.s. planes) are estimated using the full covariance matrix. The cell e.s.d.'s are taken into account individually in the estimation of e.s.d.'s in distances, angles and torsion angles; correlations between e.s.d.'s in cell parameters are only used when they are defined by crystal symmetry. An approximate (isotropic) treatment of cell e.s.d.'s is used for estimating e.s.d.'s involving l.s. planes.
Refinement. Refinement of *F*^2^ against ALL reflections. The weighted *R*-factor *wR* and goodness of fit *S* are based on *F*^2^, conventional *R*-factors *R* are based on *F*, with *F* set to zero for negative *F*^2^. The threshold expression of *F*^2^ > σ(*F*^2^) is used only for calculating *R*-factors(gt) *etc*. and is not relevant to the choice of reflections for refinement. *R*-factors based on *F*^2^ are statistically about twice as large as those based on *F*, and *R*- factors based on ALL data will be even larger.

### Fractional atomic coordinates and isotropic or equivalent isotropic displacement parameters (Å^2^)

**Table:**

	*x*	*y*	*z*	*U*_iso_*/*U*_eq_	
C1	0.66574 (17)	0.75233 (16)	0.97300 (13)	0.0626 (4)	
C2	0.62260 (15)	0.70881 (13)	0.89685 (11)	0.0515 (4)	
C3	0.56205 (15)	0.65629 (13)	0.80251 (11)	0.0537 (4)	
H3A	0.4755	0.6554	0.7911	0.064*	
H3B	0.5786	0.7004	0.7509	0.064*	
C4	0.55619 (14)	0.47568 (12)	0.72216 (9)	0.0448 (3)	
C5	0.46132 (14)	0.51032 (13)	0.64241 (10)	0.0475 (3)	
H5	0.4257	0.5822	0.6419	0.057*	
C6	0.42044 (13)	0.43671 (13)	0.56392 (10)	0.0466 (3)	
H6	0.3566	0.4598	0.5107	0.056*	
C7	0.47225 (12)	0.32897 (12)	0.56250 (10)	0.0413 (3)	
C8	0.56578 (14)	0.29515 (13)	0.64418 (11)	0.0496 (4)	
H8	0.6010	0.2230	0.6450	0.059*	
C9	0.60697 (15)	0.36650 (13)	0.72363 (11)	0.0525 (4)	
H9	0.6684	0.3421	0.7781	0.063*	
C10	0.42741 (13)	0.25699 (12)	0.47322 (10)	0.0437 (3)	
C11	0.50561 (13)	0.16440 (12)	0.45563 (10)	0.0440 (3)	
H11	0.5799	0.1498	0.5016	0.053*	
C12	0.47078 (13)	0.10202 (11)	0.37496 (10)	0.0433 (3)	
H12	0.3920	0.1139	0.3355	0.052*	
C13	0.54076 (12)	0.01680 (11)	0.34013 (9)	0.0385 (3)	
C14	0.49568 (12)	−0.01780 (11)	0.24300 (10)	0.0411 (3)	
H14	0.4206	0.0082	0.2047	0.049*	
C15	0.56300 (12)	−0.09099 (11)	0.20353 (9)	0.0384 (3)	
C16	0.67466 (12)	−0.13097 (11)	0.26139 (9)	0.0373 (3)	
C17	0.71902 (12)	−0.09784 (11)	0.35925 (9)	0.0378 (3)	
C18	0.65216 (12)	−0.02431 (11)	0.39853 (9)	0.0389 (3)	
H18	0.6814	−0.0024	0.4636	0.047*	
C19	0.41300 (14)	−0.09359 (15)	0.04783 (11)	0.0557 (4)	
H19A	0.3518	−0.1213	0.0743	0.084*	
H19B	0.3999	−0.1253	−0.0161	0.084*	
H19C	0.4094	−0.0110	0.0438	0.084*	
C20	0.73073 (19)	−0.32061 (14)	0.24124 (15)	0.0709 (5)	
H20A	0.7478	−0.3316	0.3101	0.106*	
H20B	0.7867	−0.3647	0.2186	0.106*	
H20C	0.6499	−0.3457	0.2083	0.106*	
C21	0.88002 (15)	−0.10781 (15)	0.50787 (11)	0.0560 (4)	
H21A	0.8881	−0.0254	0.5118	0.084*	
H21B	0.9580	−0.1428	0.5342	0.084*	
H21C	0.8280	−0.1327	0.5445	0.084*	
O1	0.60592 (11)	0.54149 (9)	0.80317 (7)	0.0595 (3)	
O2	0.33027 (10)	0.27793 (11)	0.41288 (9)	0.0656 (3)	
O3	0.52865 (9)	−0.12805 (9)	0.10934 (7)	0.0508 (3)	
O4	0.74235 (9)	−0.20129 (9)	0.22148 (7)	0.0471 (3)	
O5	0.82941 (9)	−0.14134 (9)	0.40858 (7)	0.0513 (3)	
H1	0.6977 (17)	0.7866 (17)	1.0333 (15)	0.081 (6)*	

### Atomic displacement parameters (Å^2^)

**Table:**

	*U*^11^	*U*^22^	*U*^33^	*U*^12^	*U*^13^	*U*^23^
C1	0.0697 (11)	0.0595 (10)	0.0548 (10)	0.0012 (8)	0.0136 (8)	−0.0162 (8)
C2	0.0608 (9)	0.0438 (8)	0.0507 (8)	0.0022 (7)	0.0183 (7)	−0.0053 (6)
C3	0.0695 (10)	0.0430 (8)	0.0446 (8)	0.0057 (7)	0.0114 (7)	−0.0059 (6)
C4	0.0563 (8)	0.0421 (7)	0.0354 (6)	0.0020 (6)	0.0133 (6)	−0.0037 (5)
C5	0.0540 (8)	0.0393 (7)	0.0469 (7)	0.0079 (6)	0.0120 (6)	−0.0060 (6)
C6	0.0440 (8)	0.0470 (8)	0.0461 (7)	0.0050 (6)	0.0098 (6)	−0.0068 (6)
C7	0.0416 (7)	0.0409 (7)	0.0447 (7)	−0.0006 (6)	0.0182 (6)	−0.0073 (6)
C8	0.0623 (9)	0.0397 (7)	0.0478 (8)	0.0110 (7)	0.0185 (7)	−0.0008 (6)
C9	0.0627 (9)	0.0499 (8)	0.0403 (7)	0.0129 (7)	0.0085 (7)	−0.0003 (6)
C10	0.0413 (7)	0.0422 (7)	0.0507 (8)	−0.0013 (6)	0.0185 (6)	−0.0099 (6)
C11	0.0436 (7)	0.0417 (7)	0.0489 (7)	0.0035 (6)	0.0176 (6)	−0.0060 (6)
C12	0.0442 (7)	0.0384 (7)	0.0506 (7)	0.0023 (6)	0.0195 (6)	−0.0047 (6)
C13	0.0439 (7)	0.0328 (6)	0.0431 (7)	−0.0010 (5)	0.0198 (6)	−0.0042 (5)
C14	0.0397 (7)	0.0394 (7)	0.0439 (7)	0.0017 (6)	0.0125 (6)	−0.0047 (5)
C15	0.0436 (7)	0.0360 (6)	0.0361 (6)	−0.0032 (5)	0.0130 (5)	−0.0056 (5)
C16	0.0440 (7)	0.0315 (6)	0.0398 (6)	0.0017 (5)	0.0179 (5)	−0.0037 (5)
C17	0.0442 (7)	0.0322 (6)	0.0377 (6)	0.0022 (5)	0.0134 (5)	0.0011 (5)
C18	0.0494 (8)	0.0360 (6)	0.0334 (6)	0.0003 (5)	0.0158 (5)	−0.0031 (5)
C19	0.0524 (9)	0.0623 (10)	0.0454 (8)	−0.0042 (7)	0.0047 (7)	−0.0069 (7)
C20	0.0933 (14)	0.0419 (9)	0.0845 (13)	0.0139 (9)	0.0375 (11)	−0.0095 (8)
C21	0.0565 (9)	0.0602 (9)	0.0436 (8)	0.0056 (8)	0.0038 (7)	−0.0048 (7)
O1	0.0829 (8)	0.0467 (6)	0.0391 (5)	0.0131 (5)	0.0040 (5)	−0.0076 (4)
O2	0.0459 (6)	0.0703 (8)	0.0715 (8)	0.0109 (5)	0.0043 (5)	−0.0307 (6)
O3	0.0498 (6)	0.0608 (6)	0.0380 (5)	0.0056 (5)	0.0077 (4)	−0.0136 (4)
O4	0.0528 (6)	0.0460 (6)	0.0470 (5)	0.0071 (4)	0.0218 (4)	−0.0092 (4)
O5	0.0522 (6)	0.0549 (6)	0.0414 (5)	0.0158 (5)	0.0063 (4)	−0.0068 (4)

### Geometric parameters (Å, °)

**Table:**

C1—C2	1.173 (2)	C13—C14	1.3960 (18)
C1—H1	0.92 (2)	C13—C18	1.3965 (19)
C2—C3	1.460 (2)	C14—C15	1.3890 (18)
C3—O1	1.4248 (18)	C14—H14	0.9300
C3—H3A	0.9700	C15—O3	1.3628 (15)
C3—H3B	0.9700	C15—C16	1.3918 (19)
C4—O1	1.3670 (16)	C16—O4	1.3758 (15)
C4—C5	1.388 (2)	C16—C17	1.4001 (17)
C4—C9	1.395 (2)	C17—O5	1.3610 (16)
C5—C6	1.3810 (19)	C17—C18	1.3862 (17)
C5—H5	0.9300	C18—H18	0.9300
C6—C7	1.3901 (19)	C19—O3	1.4242 (18)
C6—H6	0.9300	C19—H19A	0.9600
C7—C8	1.394 (2)	C19—H19B	0.9600
C7—C10	1.4886 (18)	C19—H19C	0.9600
C8—C9	1.375 (2)	C20—O4	1.428 (2)
C8—H8	0.9300	C20—H20A	0.9600
C9—H9	0.9300	C20—H20B	0.9600
C10—O2	1.2220 (18)	C20—H20C	0.9600
C10—C11	1.4784 (18)	C21—O5	1.4256 (17)
C11—C12	1.3239 (19)	C21—H21A	0.9600
C11—H11	0.9300	C21—H21B	0.9600
C12—C13	1.4632 (17)	C21—H21C	0.9600
C12—H12	0.9300		
			
C2—C1—H1	178.4 (12)	C15—C14—C13	120.02 (12)
C1—C2—C3	176.70 (18)	C15—C14—H14	120.0
O1—C3—C2	108.27 (12)	C13—C14—H14	120.0
O1—C3—H3A	110.0	O3—C15—C14	124.77 (12)
C2—C3—H3A	110.0	O3—C15—C16	115.37 (11)
O1—C3—H3B	110.0	C14—C15—C16	119.86 (12)
C2—C3—H3B	110.0	O4—C16—C15	119.71 (11)
H3A—C3—H3B	108.4	O4—C16—C17	120.13 (12)
O1—C4—C5	124.63 (13)	C15—C16—C17	120.15 (11)
O1—C4—C9	115.27 (12)	O5—C17—C18	124.90 (11)
C5—C4—C9	120.09 (13)	O5—C17—C16	115.08 (11)
C6—C5—C4	119.20 (13)	C18—C17—C16	120.01 (12)
C6—C5—H5	120.4	C17—C18—C13	119.80 (11)
C4—C5—H5	120.4	C17—C18—H18	120.1
C5—C6—C7	121.68 (13)	C13—C18—H18	120.1
C5—C6—H6	119.2	O3—C19—H19A	109.5
C7—C6—H6	119.2	O3—C19—H19B	109.5
C6—C7—C8	118.08 (12)	H19A—C19—H19B	109.5
C6—C7—C10	118.53 (13)	O3—C19—H19C	109.5
C8—C7—C10	123.37 (12)	H19A—C19—H19C	109.5
C9—C8—C7	121.21 (13)	H19B—C19—H19C	109.5
C9—C8—H8	119.4	O4—C20—H20A	109.5
C7—C8—H8	119.4	O4—C20—H20B	109.5
C8—C9—C4	119.68 (14)	H20A—C20—H20B	109.5
C8—C9—H9	120.2	O4—C20—H20C	109.5
C4—C9—H9	120.2	H20A—C20—H20C	109.5
O2—C10—C11	120.41 (12)	H20B—C20—H20C	109.5
O2—C10—C7	120.54 (12)	O5—C21—H21A	109.5
C11—C10—C7	118.94 (12)	O5—C21—H21B	109.5
C12—C11—C10	120.54 (13)	H21A—C21—H21B	109.5
C12—C11—H11	119.7	O5—C21—H21C	109.5
C10—C11—H11	119.7	H21A—C21—H21C	109.5
C11—C12—C13	128.12 (13)	H21B—C21—H21C	109.5
C11—C12—H12	115.9	C4—O1—C3	117.30 (11)
C13—C12—H12	115.9	C15—O3—C19	117.78 (11)
C14—C13—C18	120.15 (12)	C16—O4—C20	112.95 (12)
C14—C13—C12	117.40 (12)	C17—O5—C21	117.28 (11)
C18—C13—C12	122.34 (12)		
			
O1—C4—C5—C6	178.74 (14)	C13—C14—C15—C16	0.9 (2)
C9—C4—C5—C6	−1.7 (2)	O3—C15—C16—O4	1.05 (18)
C4—C5—C6—C7	−0.3 (2)	C14—C15—C16—O4	−178.49 (12)
C5—C6—C7—C8	1.6 (2)	O3—C15—C16—C17	179.73 (12)
C5—C6—C7—C10	−176.90 (14)	C14—C15—C16—C17	0.2 (2)
C6—C7—C8—C9	−0.8 (2)	O4—C16—C17—O5	−0.82 (18)
C10—C7—C8—C9	177.62 (14)	C15—C16—C17—O5	−179.50 (12)
C7—C8—C9—C4	−1.3 (2)	O4—C16—C17—C18	178.14 (12)
O1—C4—C9—C8	−177.92 (14)	C15—C16—C17—C18	−0.53 (19)
C5—C4—C9—C8	2.5 (2)	O5—C17—C18—C13	178.66 (12)
C6—C7—C10—O2	−17.5 (2)	C16—C17—C18—C13	−0.19 (19)
C8—C7—C10—O2	164.17 (15)	C14—C13—C18—C17	1.3 (2)
C6—C7—C10—C11	158.74 (13)	C12—C13—C18—C17	−174.75 (12)
C8—C7—C10—C11	−19.6 (2)	C5—C4—O1—C3	−4.2 (2)
O2—C10—C11—C12	−0.9 (2)	C9—C4—O1—C3	176.24 (14)
C7—C10—C11—C12	−177.07 (13)	C2—C3—O1—C4	177.33 (13)
C10—C11—C12—C13	172.41 (13)	C14—C15—O3—C19	−3.0 (2)
C11—C12—C13—C14	−165.06 (14)	C16—C15—O3—C19	177.52 (12)
C11—C12—C13—C18	11.1 (2)	C15—C16—O4—C20	−98.73 (16)
C18—C13—C14—C15	−1.6 (2)	C17—C16—O4—C20	82.59 (17)
C12—C13—C14—C15	174.59 (12)	C18—C17—O5—C21	−0.5 (2)
C13—C14—C15—O3	−178.61 (13)	C16—C17—O5—C21	178.43 (12)

### Hydrogen-bond geometry (Å, °)

**Table:**

Cg1 is the centroid of the C13–C18 ring.

**Table:**

*D*—H···*A*	*D*—H	H···*A*	*D*···*A*	*D*—H···*A*
C12—H12···O2	0.93	2.42	2.7710 (19)	102
C19—H19A···O2^i^	0.96	2.48	3.396 (2)	161
C20—H20B···Cg1^ii^	0.96	2.61	3.487 (2)	152

Symmetry codes:  (i) −*x*+1/2, *y*−1/2, −*z*+1/2; (ii) −*x*+3/2, *y*−1/2, −*z*+1/2.
